# Dietary Fibre Intake in Chile: 13 Years after the Last National Report

**DOI:** 10.3390/nu15173671

**Published:** 2023-08-22

**Authors:** Carla Guzmán-Pincheira, Jonathan Espinoza, Samuel Durán-Agüero, Ana María Obregón, Fabiola Fuentealba

**Affiliations:** 1 Escuela de Nutrición y Dietética, Facultad de Ciencias para el Cuidado de la Salud, Universidad San Sebastián, Campus Concepción, Concepción 4080871, Chile; carla.guzman@uss.cl (C.G.-P.); jonathan.espinoza@uss.cl (J.E.); samuel.duran@uss.cl (S.D.-A.); ana.obregon@uss.cl (A.M.O.); 2 Vicerectoría de Vinculación con el Medio, Universidad San Sebastián, Concepción 4080872, Chile

**Keywords:** dietary fibre, food frequency questionnaire, health effects

## Abstract

Background: The objective is to provide updated data on the intake of total dietary fibre in the population residing in Chile and to identify food sources that contribute most to its intake, as well as its association with different sociodemographic and nutritional status-related determinants. Methods: In this descriptive cross-sectional study, a nationwide survey was applied to determine fibre intake using an instrument that has been previously validated in the resident population of Chile. Results: The sample consisted of a total of 1761 participants. Dietary fibre intake was 12.8 ± 7.1 g/day for the total population, and 90% of participants did not meet the recommendation, with no differences in consumption by sex, geographical area, and the urban/rural population. A lower consumption was found among participants with overweight and obesity. According to food groups, bread was the major contributor to fibre intake, providing 4.39 ± 3.05 g/day, followed by cereals (2.26 ± 2.80 g/day) and vegetables (1.85 ± 1.59 g/day). Conclusions: 90% of the population consume less fibre than recommended, and bread is the main food source; these data are critical for the development of strategies that are aimed at changing habits in order to improve diet quality.

## 1. Introduction

Currently, there is a long research history on dietary fibre (DF) and its benefits on human health [1]; however, in some countries, there is still a lack of information on the intake of this nutrient, particularly in Latin America, as a result of the limited availability of data sets, which combined with the fact that the Western dietary pattern is characterised by low fibre intake, creates a need to conduct food frequency questionnaires with the aim of quantifying the total DF intake, knowing the food sources that contribute most to its consumption, and eventually, defining medium and long-term strategies to increase its daily intake [2].

DF has been defined in terms of its structure as a group of carbohydrates resistant to digestion and absorption in the small intestine [3], with its most well-known classification related to water hydration capacity, comprising soluble dietary fibre (SDF) and insoluble dietary fibre (IDF). The former includes pectins, gums, and mucilages, present, for instance, in apples, pears, citrus fruits, carrots, peas, cucumber, celery, and wheat bran [4], while the latter includes cellulose, hemicellulose, and lignin, which are present in foods such as dried fruits, pulses, whole wheat, barley, and roots [5,6]. When comparing their physiological effects, SDF forms colloidal solutions in the intestine, slows down digestion, and provides a prolonged feeling of satiety, unlike IDF, which accelerates intestinal transit, increases stool bulk, and acts as a laxative [7]. Epidemiological studies have consistently demonstrated that a higher intake of dietary fibre is associated with a lower risk of depression [8,9,10], ulcerative colitis [11], and cardiovascular disease [12,13]; greater weight loss in overweight/obesity [14]; changes in blood glucose levels [15,16]; an increased the relative abundance of *Bifidobacterium* [4,17]; and a lower risk of all types of cancer [17,18], especially breast [19,20], colorectal [21], pancreatic [22], and bladder cancers [23]. Moreover, in individuals with hypertension or cardiovascular disease, DF reduces the total cholesterol, low-density lipoprotein (LDL) cholesterol, systolic and diastolic blood pressure [24], while in critically ill patients, defined as those suffering from organic instability and/or who are in a life-threatening situation, DF may improve the gut barrier function, modulate gut microbiota, decrease systemic inflammation and improve clinical outcomes [25], as well as decreasing uremic toxin levels in chronic kidney disease [26].

No unique consensus has been reached on the recommendation for dietary fibre intake. Based on the available scientific evidence, the European Food Safety Authority (EFSA), the Food and Agriculture Organization of the United Nations (FAO), and the World Health Organization (WHO) set out a recommended intake of 25 g per day for adults and indicated that this recommendation could be achieved through the consumption of food sources such as fruits, fresh vegetables, potatoes, whole grains, pulses, nuts, and seeds [27]. Despite the information aforementioned, DF intake does not exceed 20 g per day at the global level. In this context, in 2015, the Scientific Advisory Committee on Nutrition in the United Kingdom recommended increasing this intake to 30 g per day; however, only 9% of adults in the United Kingdom reached this goal [28].

According to data from the National Health and Nutrition Examination Survey (NHANES) 2017–2018, the mean daily fiber intake in the United States was 16.2 g, which represented a decrease of approximately 0.3 g compared to the 16.5 g reported in 2015–2016 [29,30]; nevertheless, it is still below the recommendation, and moreover, there is scant detailed information on the types of fibre and, particularly, on the food groups that are a source of DF consumed by the population. Despite the high accordance on the importance of having updated information on DF intake, this situation was repeated in Latin America, so much so that the latest data on dietary fibre intake in Chile is that reported 13 years ago by the National Food Consumption Survey (ENCA, as its acronym in Spanish) 2010, where the average was 12.5 g/day and 11.5 g/day in men and women, respectively.

Based on the above, the main objective of this study was to provide updated data on the intake of total dietary fibre in the population residing in Chile to identify the main food sources that contribute most to its intake, as well as its association with different sociodemographic and nutritional status-related determinants.

## 2. Materials and Methods

### 2.1. Study Design and Population

A descriptive cross-sectional study was conducted in 1761 adults aged 18 years or older residing in Chile in the period between December 2022 and May 2023. Subjects who had been diagnosed with severe liver disease, kidney failure, gastric or colon cancer, food allergies and intolerances, celiac disease, Crohn’s disease, ulcerative colitis, pregnancy, and/or those with diseases that cause an alteration in consumption patterns (i.e., irritable bowel syndrome) were excluded. A non-probabilistic sampling method was used, with an open call being addressed to the community through virtual and/or social platforms. The recruitment process included the initial screening of participants and obtaining informed consent. A total of 1761 participants were recruited, and the following sociodemographic and nutritional variables were collected: sex (men and women), age (years), occupation (dependent worker, freelance worker, student, unemployed, housekeeper, and retired), macrozone of residence (North, Center, South, and Metropolitan), place of residence (urban and rural), body mass index category (kg/m^2^); in addition, the dietary fibre intake short food frequency questionnaire (DF-FFQ) was applied.

### 2.2. Determination of Dietary Fiber Intake

For the determination of dietary fibre intake, we used an instrument that had been previously validated in the Chilean resident population (DF-FFQ) [31], which was designed for online application, thus allowing data collection in specific population groups who are difficult to access due to the geographical characteristics of the country. The questionnaire includes 59 foods that contain fibre classified into 5 food groups (fruits, vegetables, bread-cereals, dried fruits, and pulses), with consumption frequencies ranging from “never” to “6 or more times per day”; in order to reduce reporting bias in relation to the size of food servings, the questionnaire contained representative images of each group obtained from the Atlas Fotográfico de Preparaciones Típicas Chilenas [Photographic Atlas of Typical Chilean Preparations]. As described in the pilot study of this instrument, bread is the food item that contributes most to total fibre intake among the resident population of Chile; therefore, it was decided that the cereals and bread groups should be subdivided, estimating fibre intake independently for both types of food. Fiber consumption was quantified using the Table of the Chemical Composition of Chilean Food [32], and was subsequently calculated using a scoring sheet. The total dietary fibre intake of a participant was calculated as the sum of the average amount of fibre consumed from each food group in relation to the number of consumed servings.

### 2.3. Ethical Aspects

This research was conducted in accordance with the Declaration of Helsinki regarding work involving human beings [33] and the Council for International Organizations of Medical Sciences (CIOMS) guidelines [34]. The protocol of this study was approved by the Scientific Ethics Committee of the Universidad San Sebastián (No. 5–22). All participants were informed of their participation in the study, and their informed consent was obtained.

### 2.4. Statistical Analysis 

Statistical analysis was performed with the IBM SPSS Statistics V21 software (Armonk, NY, USA, 2012). Descriptive statistics for quantitative variables were described with the mean and standard deviation, while categorical variables were described with frequencies and percentages. Data were tested for normality using the Kolmogorov–Smirnov test, and the Mann–Whitney or Kruskal–Wallis tests were used, as appropriate, to evaluate the differences between the groups, while the Spearman test was used to determine the correlation between variables. A *p*-value of <0.05 was considered statistically significant for all tests.

## 3. Results

### 3.1. Sociodemographic Characteristics, Nutritional Status and Dietary Fiber Intake

Participants were divided into two groups according to their sex (men and women). The frequencies and percentages of the general characteristics of the population are shown in Table 1. The sample consisted of a total of 1761 participants, distributed 65.6% of women and 34.1% of men, while 0.34% preferred not to answer. Age was categorised into groups as follows: youths (18–29 years old), adults (30–59 years old), and older adults (≥60 years old), with each group accounting for 44.1%, 50.0%, and 5.9% of the total sample, respectively. The population was mostly concentrated in the Central (44.3%) and Metropolitan (38.5%) macrozones, residing in urban areas (92.6%). The occupations of the participants were in decreasing order: dependent workers (46.3%), students (34.9%), and freelance workers (10.8%). With respect to nutritional status, most participants presented malnutrition by excess according to the body mass index (BMI) classification, with overweight reaching 34.8% and obesity at 18.7%, while 44.7% of the sample had a normal nutritional status with a BMI between 18.5–24.9 kg/m^2^ for adults and 23.1–27.9 kg/m^2^ for adults over 60 years of age. 

The mean dietary fibre intake was 12.8 ± 7.1 g/day for the total population, which is similar to that reported by the previously published pilot study, where a mean intake of 12.3 ± 6.9 g/day [31] was observed. When comparing the intake by sex, mean intakes of 12.7 ± 6.8 g/day and 12.8 ± 7.6 g/day were observed in women and men, respectively, with no statistically significant difference between the two groups (*p* = 0.467). The mean dietary fibre intake is presented according to general characteristics in Table 2. Higher mean fibre intakes were found in the north of the country (13.6 ± 6.3 g/day), with the intake being 8% higher than that reported in the area of lowest consumption, represented by the southern area, with a mean intake of 12.5 ± 6.9 g/day, showing no significant statistical difference (*p* = 0.429); a similar situation was observed when comparing fibre consumption among the population residing in urban areas (12.8 ± 7.1 g/day) versus rural areas (12.7 ± 5.6 g/day) with no significant differences noted (*p* = 0.529).

**Figure 1:**
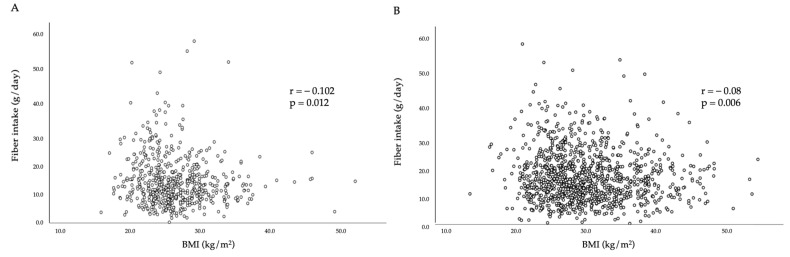
Correlation between dietary fibre intake and body mass index (BMI) in men (**A**) and women (**B**). Spearman test; statistical significance *p* < 0.05.

On the other hand, a significant difference in dietary fibre intake was observed in the 18–29 and 30–59 age groups (*p* = 0.016), which, when separated by sex, was only observed in women of the same age group (*p* = 0.017); when analysed by occupation, among the participants, a difference was observed specifically between those who were dependent workers and students (*p* = 0.020). Additionally, a difference in fibre intake was noted depending on the nutritional status of the participants, with a lower consumption observed in those who had malnutrition by excess, specifically overweight and obesity, with a mean intake of 12.1 ± 7.0 g/day and 12.2 ± 6.1 g/day, respectively, while the intake of participants with normal nutritional status was 13.6 ± 7.4 g/day (*p* < 0.001). Considering the above facts, it was decided to analyse the total DF intake with respect to BMI, with a weak negative association observed, both in men (r = −0.102; *p* = 0.012) and women (r = −0.08; *p* = 0.006), with a statistically significant difference found in both groups (Figure 1).

### 3.2. Main Food Sources Contributing to Dietary Fiber

**Figure 2:**
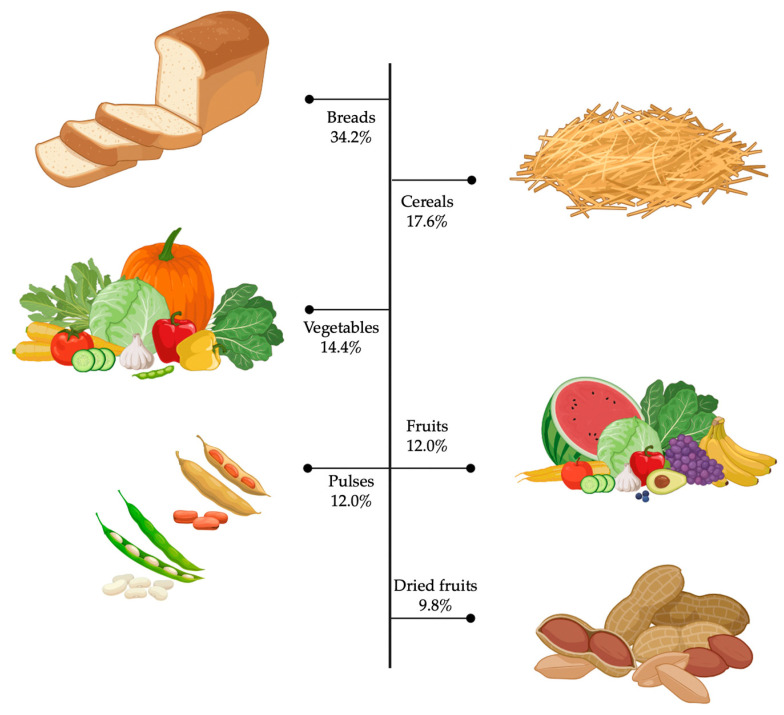
Total dietary fibre percentage distribution (g/day) by food group in the total sample (n = 1761).

**Figure 3:**
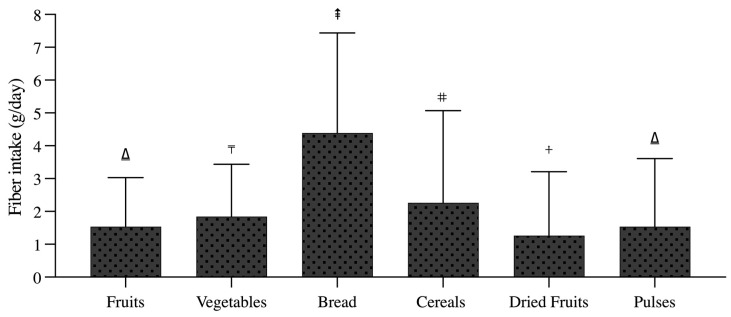
Mean total dietary fibre intake of the main food groups in the total sample (n = 1761). Dietary fibre intake is expressed in grams (g). Comparisons between groups were performed with the Kruskal–Wallis test. Different symbols represent statistical significance *p* < 0.05.

**Figure 4:**
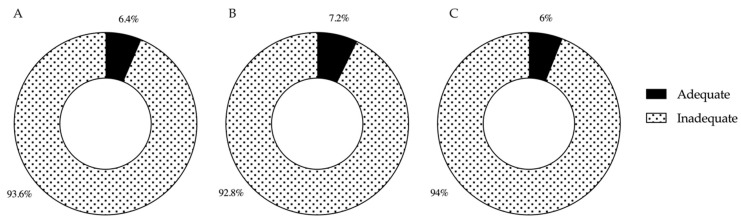
Compliance rate with the recommended minimum intake of dietary fibre according to the European Food Safety Authority (EFSA), Food and Agriculture Organization of the United Nations (FAO), and World Health Organization (WHO). Data are presented as a percentage (%) for (**A**) Total population; (**B**) Men; (**C**) Women.

It can be noted that the bread food group is the major contributor to total fibre intake, in a transversal manner, accounting for 34.2% and representing the most consumed food item by the residents of the national territory, with an average of seven daily servings. The contribution, in decreasing order, corresponds to bread (4.39 ± 3.05 g/day), cereals (2.26 ± 2.80 g/day), vegetables (1.85 ± 1.59 g/day), fruits (1.54 ± 1.49 g/day), pulses (1.54 ± 2.07 g/day), and dried fruits (1.26 ± 1.94 g/day) (Figure 2), with statistically significant differences between groups (*p* < 0.05), except for fruits versus pulses (*p* > 0.05) (Figure 3). In addition to the above, the prevalence of “inadequate intake”, considering a recommended minimum intake of 25 g/day established by the European Food Safety Authority (EFSA), the Food and Agriculture Organization of the United Nations (FAO) and the World Health Organization (WHO), is observed among more than 90% of the population, both men and women (Figure 4), which stresses the need for the development of strategies aimed at increasing the intake of this nutrient. 

Table 3 describes the dietary fibre intake by food groups and its comparison across variables of the total population. Specifically, in the case of fiber derived from bread, all age groups, including youths, adults, and older adults, had a similar intake with no differences being found (*p* = 0.422); a similar situation was observed in participants from the different macrozones (*p* = 0.120) and areas of residence, both urban and rural, though. the latter group had a slightly higher intake (4.36 ± 3.05 versus 4.65 ± 3.06 g/day). However, no statistically significant differences were found (*p* = 0.219). When comparing the consumption of this food group by sex, it was observed to be more greatly consumed in men (4.69 ± 3.29 g/day) than in women (4.23 ± 2.90 g/day), with a significant difference between the two sexes (*p* = 0.021).

According to the results obtained in this study, it was observed that the consumption of dietary fibre from fruit and vegetables is significantly higher in older people and in women (*p* < 0.05). Likewise, with regard to the exclusive analysis of fibre ingested from fruit, significant differences were found in consumption according to occupation, with dependent workers and retired people showing statistical differences (*p* = 0.043). In addition, it was found that individuals with a normal nutritional status showed higher consumption of fibre through fruit intake compared to those who were overweight (*p* = 0.002) or obese (*p* < 0.001). These findings suggest the importance of promoting higher dietary fibre intake, especially from sources such as fruits and vegetables, to improve health and well-being in different population groups. Similarly, it was found that participants with nutritional status of obesity showed an increase in bread consumption, favouring a greater intake of fibre that reached 5.17 ± 3.29 g/day (*p* < 0.001).

This study shows that dietary fibre intake from cereals varies significantly across different demographic groups. Young people show a higher intake of cereal fibre (*p* < 0.001), as do students relative to homeowners, self-employed and dependent workers (*p* < 0.001). In addition, people with normal nutritional status were found to consume more cereal fibre than those with excess malnutrition, such as overweight or obesity. Regarding fibre consumption from nuts, higher consumption was found in people over 60 years of age (*p* = 0.001) and among students and the self-employed (*p* = 0.041). In addition, it was found that those with normal nutritional status consumed more fibre from nuts (*p* < 0.001) and pulses (*p* = 0.014) compared to those with obesity. 

## 4. Discussion

The aim of this research study was to assess dietary fibre intake in the resident population of Chile 13 years after the last national report and to know the main food sources contributing to its intake on the basis of the application of a previously validated short food frequency questionnaire on high-fibre foods [31]. A total of 1761 subjects residing in the national territory participated in the study, who lived mainly in urban areas, with the Centre and Metropolitan macrozones being the most representative. The sample consisted mostly of women, who represented 65.6% of the total population, which is consistent with the population density data of the country [35]. The main result of this research revealed that dietary fibre intake did not meet the daily intake recommendations proposed by national and international bodies, such as the Food and Agriculture Organization of the United Nations (FAO), the World Health Organization (WHO) [36] and the European Food Safety Authority (EFSA) [27], and that bread is the main food source contributing to dietary fibre intake, with fibre contribution from bread exceeding that of cereals, pulses, fruits, and vegetables.

The total fibre intake for the resident population of Chile was 12.4 g/day: a result similar to that found in the pilot study for the validation of the dietary fibre intake short food frequency instrument, which averaged a total of 12.3 ± 6.9 g/day [31], which is equivalent to approximately 50% of the requirements for this nutrient. The intake of fibre should reach 18–38 g/day for adults since it is associated with beneficial effects such as reduced risk of stroke [36]; improved metabolic control in overweight, obesity, type 2 diabetes mellitus, and cardiovascular diseases [12,13,15]; the recovery of the intestinal transit [37]; and the reduced risk of certain types of cancer, due to its functional properties such as solubility, viscosity, fermentability, hydration capacity, and its capacity to adsorb organic molecules [38].

Considering our knowledge of the role of fibre in health, recommendations have been made to increase the intake of this nutrient through the diet, so much so that in Europe, there have been statements on health properties in relation to intestinal function that have classified foods according to DF content, with “high content” foods being those that provide at least 6 g of DF per 100 g of food, or 3 g of DF per 100 kcal [39], while, in Chile, Article 120 of the Food Health Regulations states in a nutritional description that a good source of fibre is a dietary source that contains between 10% and 19.9% of the daily recommendations [40].

When comparing the results found in this study with those described by other researchers, it is noted that the insufficient intake of fibre is a reality common to several countries in this region, such as Brazil (15.7 g/day) [41,42], Argentina (9.3 g/day) [43] or Mexico (17 g/day) [44]; these data sharply contrast with those reported in countries such as Sweden and Norway that show dietary fibre intake ranging from 19 to 24 g/day [1]. In Europe, studies have quantified fibre intake using national food surveys, which have shown a higher consumption in men compared to women, with 21 and 19 g/day, respectively, with the lowest intakes reported in the United Kingdom (15 g in men versus 13 g in women) and the highest in Germany (27 g in men versus 25 g in women) [45], while among the Canadian population, fibre intake is higher in men than in women, with 18.2 g and 16.3 g, respectively [46].

It is worth noting that, according to that reported in our study, the greatest fibre contribution is provided by the consumption of white bread, exceeding the contribution made by groups of cereals, pulses, fruits, vegetables, and dried fruits, which is in accordance with the bread consumption pattern reported by the Federación Nacional de Industriales Panaderos de Chile [National Federation of Bakers, FECHIPAN, as its Spanish acronym], who indicate that the population residing in the national territory are the second largest consumers of bread worldwide after Turkey, with an annual consumption of 98 kg per capita [47]. The aforementioned information, together with previous research on Chilean elderly people that showed bread, irrespective of the type of flour used in its preparation, to be a source that provides significant amounts of other critical nutrients, such as iron, zinc, calcium, folic acid, pantothenic acid, magnesium and selenium [48], is interesting. This is because the potential transition from white bread to whole wheat bread, which provides a greater dietary fibre contribution, may have a positive impact on the health of the population. In this context, it has been observed that cereal products are the largest source of fibre, contributing between 32–33% of the intake of DF in the United States and Spain and between 48–49% in Ireland and the Netherlands, while in those studies that offered a greater breakdown of cereal sources, bread was the main source accounting for 11–30% of DF, followed by vegetables (12–21%) and fruits (8–23%), showing a great deal of variation, probably due to the climatic conditions of growth in the different regions and different cultures of food consumption [1]. The above is relevant since a distinction has been made between the effects of different sources of dietary fiber on the presence of diseases, so much so that fiber from cereals, pulses, fruits, or vegetables has been associated with a reduced risk of colorectal cancer and recurrent adenoma [49], with the laxative effect of insoluble fibre [50], a reduction in faecal transit time and the increase in stool bulk [51] being the possible mechanism behind this fact in addition to particular effects on the composition of gut microbiota [52].

Moreover, different sources of dietary fibre may present particular characteristics which have been previously described, such as a reduction in deaths attributed to chronic diseases [51,53,54], which could be explained by the fact that high-fibre diets, including cereal dietary fibre are also high in folate, selenium, magnesium, copper, phenolic acids, lignans, and phytochemicals [55], which could play a role in the protective association of cereal fibre with mortality, and, in turn, could lead to lower levels of inflammatory markers, including the C-reactive protein (CRP) and tumour necrosis factor-alpha (TNFα) receptor 2 [56]. Furthermore, high consumption of dietary fibre from cereals has been shown to be associated with a lower risk of type 2 diabetes [50], an improved postprandial glycaemic response, and increased sensitivity to insulin [57]; this possible mechanism is associated with the interference of cereal fibres with the absorption and digestion of dietary protein and the modulation of the metabolic signature of amino acids by inhibiting the activation of the rapamycin/S6 kinase 1 signalling pathway [58]. In addition, the present study shows that dietary fibre intake in Chile from food sources such as fruits, vegetables, and legumes is at inadequately low levels. This situation raises the possibility of deficiencies in other nutrients due to the low amount of these foods consumed in the diet. This reality is in line with similar findings reported in studies conducted in other countries, which have also observed a trend towards insufficient levels of dietary fibre related to the limited consumption of plant foods. It is essential to address this situation at a global level to promote a balanced diet and ensure the adequate intake of essential nutrients for optimal health and well-being [41,44].

When comparing the variables described in our research, such as place of residence and sex, no significant differences were observed in fibre consumption, except in the nutritional status variables, where a lower consumption of DF was observed in the population diagnosed with obesity. In this sense, one way to improve malnutrition due to excess indicators in the Chilean population could be through the consumption of foods that are rich in fibre or foods supplemented with DF since it has been shown that an adequate intake of this nutrient plays an important role in the prevention and treatment of malnutrition due to excess [59]. Current scientific evidence indicates that there is a greater reduction in body weight and waist circumference [60] and a better fat distribution ratio for visceral and total fat mass in populations where the carbohydrate quality has been improved, mainly due to the incorporation of dietary fibre and the whole grain/total grain ratio, which has also been associated with the stabilisation of glycaemic control [15]. This is explained by the physicochemical and physiological properties of LD, such as water retention capacity, hydration, oil retention capacity, cholesterol absorption capacity, and good glucose absorption capacity, reducing its plasma levels [61]. Significant differences were also found in the age variable, where a higher consumption of fibre from fruit and vegetables was observed in older people and in women. With regard to the elderly and fibre consumption, our results and the scientific evidence indicate that consumption is below what is recommended by international organisations [62,63,64]; therefore, we should encourage the increased consumption of vegetable foods, fruit, and cereals that are rich in dietary fibre, as it has been shown that a good intake of this nutrient in the elderly can have positive effects on improving cognitive function, preventing depression [65,66], enhancing effects on muscle quality and function, and improving and maintaining occlusal strength [67]. All of the above can be associated with fibre’s physicochemical and functional characteristics, especially those related to maintaining the balance of the intestinal microbiota [68], preventing a reduction in biodiversity and the overexpression of pathobionts. In the same way, significant differences were observed in the occupation variable, showing a higher consumption of fibre from fruit in dependent workers and retired people. These results are similar to those obtained by other studies in which a higher consumption of fruit was observed in the dependent working population, which is mainly explained by the higher educational and socioeconomic level of the workers [69,70]. Some authors point out that this can be improved through interventions and policies that encourage the consumption of high-fibre foods, e.g., making fruit available in working environments [71,72].

In addition to the above, the mechanism by which dietary fibre can contribute to the prevention of obesity is related to a reduction in hunger and prolongation of satiety [73]. There are a number of mechanical and endocrine signals from the gastrointestinal tract that are stimulated by dietary fibre and its fermentation products which reach regions of the brain involved in the regulation of appetite and ultimately reduce food intake [73]. Fiber fermentation produces short-chain fatty acids, stimulating entero-endocrine cells to induce the secretion of glucagon-like peptide-1 (GLP-1) and peptide YY (PYY) [74]. In this regard, evidence has shown that the acute intake of the type of fibre called β-glucan (oatmeal) decreases appetite, increases satiety, and reduces total energy intake [75].

Our study provides relevant information regarding fibre intake in the country, including the use of a previously validated instrument and achieving nationwide coverage in terms of participation. However, despite successful data collection, it is important to note the cross-sectional nature of this study, and therefore, results should be analysed with caution without establishing causality between them.

Among the weaknesses of this research are the nature of the questionnaire, which was applied online, and could represent a gap at the time of covering a representative sample of the country’s inhabitants, either due to the availability of the internet, geographic area, or the age of the respondents (older people). Another weakness detected is the fact that this instrument does not distinguish between whole-grain and refined foods, generating an estimate that is not entirely accurate. The anthropometric data, body weight, and height were self-reported by the participants, which is a weakness in comparison with field data obtained by clinical staff.

## 5. Conclusions

This research showed that the prevalence of an “inadequate intake” of dietary fibre, considering a minimum intake of 25 g/day, as recommended by various organisations, exceeds 90% of the population, both in men and women, and that the intake of dietary fibre in adults living in Chile does not meet international recommendations, covering only 50% of it, with the population experiencing excess malnutrition (overweight and obesity) being the one with the lowest consumption of DF; the main food source of dietary fibre consumed by Chileans is not cereals, legumes, fruit, vegetables or nuts, but bread. It would therefore be interesting to promote the replacement of white bread with wholemeal bread, which could improve or increase the consumption of dietary fibre in the population; it is also worth mentioning that men have a higher consumption of fibre than women from the consumption of bread. From the point of view of the available variety of fibre-contributing foods, it is important to highlight that, in addition to providing DF with functional characteristics, they also provide other essential nutrients that enable the normal functioning and development of the body. The inclusion of a greater number of fibre-rich foods can help maintain dietary balance, both in terms of nutrients and energy intake. These data are crucial for the development of public health policies and behavioural change strategies to improve dietary quality, especially dietary fibre intake throughout the life cycle.

## Figures and Tables

**Table 1:** Descriptive analysis of sociodemographic variables and nutritional status (n = 1761).

Kerrypnx	Total Population		Men		Women	
	n	%	n	%	n	%
**Age**						
- 18–29 years	777	44.1	307	51.2	465	40.3
- 30–59 years	880	50.0	264	44.0	615	53.2
- ≥60 years	104	5.9	29	4.8	75	6.5
**Macrozone**						
- North	54	3.1	20	3.3	34	2.9
- Center	780	44.3	200	33.3	577	50.0
- South	249	14.1	62	10.3	186	16.1
- Metropolitan	678	38.5	318	53.0	358	31.0
**Residence**						
- Urban	1630	92.6	560	93.3	1065	92.2
- Rural	131	7.4	40	6.7	90	7.8
**Occupation**						
- Dependent worker	816	46.3	283	47.2	533	46.1
- Freelance worker	190	10.8	76	12.7	113	9.8
- Student	614	34.9	213	35.5	396	34.3
- Unemployed	49	2.8	20	3.3	29	2.5
- Housekeeper	51	2.9	0	0	51	4.4
- Retired	41	2.3	8	1.3	33	2.9
**BMI Category**						
- Underweight	32	1.8	6	1.0	26	2.3
- Normal	787	44.7	220	36.7	563	48.7
- Overweight	613	34.8	246	41.0	365	31.6
- Obesity	329	18.7	128	21.3	201	17.4

Body mass index (BMI) was considered normal at 25 kg/m^2^ or less in adults and at 28 kg/m^2^ or less in older adults. Data are presented as frequencies (n) and percentages (%) by sex.

**Table 2:** Description of total fibre intake (g/day) (n = 1761).

	Total Population		Men		Women		
	Mean	SD	Mean	SD	Mean	SD	*p* Value
**Age**							
- 18–29 years	13.3	7.4	13.4	8.1	13.3	6.9	0.591
- 30–59 years	12.3	6.9	12.2	7.1	12.4	6.8	0.789
- ≥60 years	13.5	6.0	13.9	7.2	13.3	5.5	0.848
*p* value	0.005 *		0.248		0.020 *		
**Macrozone**							
- North	13.6	6.3	11.9	5.6	14.6	6.6	0.128
- Center	12.9	7.0	13.8	7.9	12.7	6.7	0.407
- South	12.5	6.9	13.8	8.9	12.0	6.1	0.214
- Metropolitan	12.7	7.3	12.2	7.3	13.1	7.3	0.073
*p* value	0.429		0.073		0.096		
**Residence**							
- Urban	12.8	7.2	12.9	7.7	12.8	6.9	0.277
- Rural	12.7	5.7	13.1	6.0	12.5	5.6	0.700
*p* value	0.529		0.403		0.810		
**Occupation**							
- Dependent worker	12.1	6.5	11.9	6.5	12.2	6.5	0.554
- Freelance worker	13.2	7.2	12.6	7.7	13.7	6.9	0.291
- Student	13.5	7.7	13.9	8.8	13.3	7.0	0.767
- Unemployed	13.9	8.2	14.4	6.6	13.7	9.3	0.416
- Housekeeper	12.8	6.0	0	0.0	12.8	6.0	-
- Retired	13.4	7.1	16.3	8.1	12.7	6.7	0.250
*p* value	0.024 *		0.092		0.130		
**BMI Category**							
- Underweight	13.9	6.8	11.5	6.1	14.5	6.9	0.412
- Normal	13.6	7.4	14.5	8.2	13.3	7.1	0.300
- Overweight	12.1	7.0	11.9	7.7	12.2	6.6	0.188
- Obesity	12.2	6.1	12.0	5.9	12.3	6.3	0.676
*p* value	<0.001 *		0.001 *		0.050		

Data are presented as mean and standard deviation (SD). BMI: body mass index (kg/m^2^). Comparisons between categories were performed with the Mann–Whitney U-test or Kruskal–Wallis test as appropriate; (*) statistical significance *p* < 0.05.

**Table 3:** Description of fibre intake (g/day) by food groups (n = 1761).

	Fruits			Vegetables			Bread			Cereals			Dried Fruits			Pulses		
	Mean	SD	95% CI	Mean	SD	95% CI	Mean	SD	95% CI	Mean	SD	95% CI	Mean	SD	95% CI	Mean	SD	95% CI
**Age (years)**																		
- 18–29	1.51	1.49	1.40–1.61	1.83	1.58	1.71–1.94	4.51	3.19	4.28–4.73	2.77	3.20	2.54–3.00	1.14	1.89	1.01–1.27	1.55	1.90	1.42–1.69
- 30–59	1.49	1.46	1.49–1.58	1.83	1.58	1.72–1.93	4.26	2.91	4.06–4.45	1.89	2.41	1.73–2.05	1.31	1.98	1.18–1.44	1.52	2.29	1.37–1.68
- ≥60	2.15	1.62	1.83–2.46	2.10	1.59	1.79–2.41	4.56	3.03	3.97–5.15	1.59	1.89	1.22–1.96	1.63	2.01	1.24–2.02	1.41	1.17	1.18–1.64
*p* value	<0.001 *			0.025 *			0.422			<0.001 *			<0.001 *			0.523		
**Macrozone**																		
- North	1.74	1.54	1.32–2.16	2.15	1.77	1.66–2.63	4.20	2.69	3.46–4.93	2.50	2.72	1.75–3.24	1.60	2.26	0.99–2.22	1.39	1.56	0.96–1.82
- Center	1.56	1.53	1.45–1.67	1.80	1.55	1.69–1.91	4.52	3.01	4.31–4.73	2.28	2.85	2.08–2.48	1.22	1.96	1.09–1.36	1.57	1.87	1.44–1.70
- South	1.35	1.36	1.18–1.52	1.73	1.59	1.53–1.93	4.45	3.27	4.04–4.86	2.26	2.87	1.90–2.62	1.21	1.91	0.97–1.44	1.45	1.57	1.25–1.64
- Metropolitan	1.55	1.49	1.44–1.66	1.91	1.61	1.79–2.03	4.22	3.03	3.99–4.45	2.22	2.73	2.01–2.42	1.28	1.91	1.13–1.42	1.54	2.4	1.34–1.71
*p* value	0.156			0.116			0.120			0.717			0.701			0.013 *		
**Sex**																		
- Women	1.61	1.50	1.52–1.69	1.93	1.59	1.84–2.02	4.23	2.90	4.06–4.40	2.22	2.65	2.07–2.37	1.22	1.76	1.12–1.33	1.55	2.18	1.42–1.68
- Men	1.40	1.47	1.28–1.52	1.67	1.56	1.55–1.80	4.69	3.29	4.43–4.69	2.32	3.08	2.07–2.57	1.30	2.25	1.12–1.48	1.48	1.84	1.33–1.62
*p* value	0.001 *			<0.001 *			0.021 *			0.537			0.299			0.158		
**Residence**																		
- Urban	1.55	1.50	1.47–1.62	1.83	1.59	1.76–1.91	4.36	3.05	4.21–4.51	2.28	2.83	2.14–2.42	1.25	1.95	1.16–1.35	1.54	2.13	1.43–1.64
- Rural	1.37	1.32	1.14–1.60	1.94	1.48	1.68–2.19	4.65	3.06	4.12–5.18	2.00	2.47	1.58–2.43	1.25	1.89	0.92–1.57	1.45	1.16	1.24–1.65
*p* value	0.435			0.161			0.219			0.451			0.994			0.302		
**Occupation**																		
- Dependent worker	1.46	1.43	1.36–1.56	1.76	1.52	1.65–1.86	4.23	2.77	4.04–4.42	1.93	2.35	1.77–2.09	1.23	1.77	1.11–1.35	1.48	2.20	1.33–1.63
- Freelance worker	1.66	1.62	1.43–1.89	2.15	1.73	1.91–2.40	4.48	3.32	4.01–4.96	1.98	2.50	1.62–2.34	1.47	2.23	1.15–1.79	1.46	1.61	1.23–1.69
- Student	1.53	1.51	1.41–1.65	1.80	1.57	1.68–1.93	4.48	3.28	4.22–4.74	2.88	3.35	2.61–3.15	1.17	1.96	1.01–1.32	1.63	2.20	1.46–1.80
- Unemployed	1.43	1.42	1.03–1.84	2.06	1.76	1.56–2.57	5.06	3.33	4.11–6.02	2.50	3.30	1.55–3.45	1.26	2.60	0.51–2.01	1.64	1.10	1.32–1.96
- Housekeeper	1.91	1.63	1.45–2.37	2.19	1.91	1.65–2.73	4.56	2.75	3.79–5.33	1.14	1.74	0.65–1.63	1.47	2.36	0.80–2.13	1.54	1.21	1.20–1.88
- Retired	2.04	1.50	1.56–2.51	1.95	1.52	1.47–2.43	4.51	3.37	3.45–5.58	1.92	2.09	1.26–2.58	1.69	2.12	1.02–2.36	1.28	0.95	0.98–1.58
*p* value	0.021 *			0.021 *			0.669			<0.001 *			0.012 *			0.110		
**BMI Category**																		
- Underweight	1.66	1.54	1.11–2.21	2.17	1.85	1.51–2.84	4.69	4.01	3.24–6.14	2.59	2.38	1.73–3.45	1.02	1.55	0.45–1.58	1.79	1.60	1.21–2.37
- Normal	1.72	1.58	1.62–1.84	1.94	1.61	1.83–2.05	4.13	2.98	3.92–4.34	2.65	2.97	2.44–2.86	1.48	2.14	1.33–1.63	1.65	2.29	1.49–1.81
- Overweight	1.41	1.37	1.29–1.52	1.73	1.50	1.61–1.85	4.27	2.87	4.05–4.50	2.05	2.77	1.83–2.27	1.13	1.81	0.99–1.28	1.50	2.09	1.33–1.66
- Obesity	1.31	1.42	1.15–1.46	1.79	1.63	1.61–1.97	5.17	3.29	4.81–5.52	1.66	2.31	1.41–1.91	0.95	1.65	0.77–1.13	1.30	1.40	1.15–1.45
*p* value	<0.001 *			0.053			<0.001 *			<0.001 *			<0.001 *			0.011 *		

Data are presented as the mean, standard deviation (SD), minimum, and maximum. Comparisons between categories were performed with the Mann–Whitney U-test or Kruskal–Wallis test as appropriate; (*) statistical significance *p* < 0.05.

## Data Availability

Not applicable.
